# Regulatory mechanisms of RNA function: emerging roles of DNA repair enzymes

**DOI:** 10.1007/s00018-014-1562-y

**Published:** 2014-02-05

**Authors:** Laure Jobert, Hilde Nilsen

**Affiliations:** 1 Division of Medicine, Department of Clinical Molecular Biology, Akershus University Hospital, Nordbyhagen, 1478 Lørenskog, Norway; 2 Department of Clinical Molecular Biology, Faculty of Medicine, Institute of Clinical Medicine, University of Oslo, Blindern, P.O.Box 1171, 0318 Oslo, Norway

**Keywords:** Base excision repair, SMUG1, APE1, RNA quality control, RNA editing, RNA epigenetics

## Abstract

The acquisition of an appropriate set of chemical modifications is required in order to establish correct structure of RNA molecules, and essential for their function. Modification of RNA bases affects RNA maturation, RNA processing, RNA quality control, and protein translation. Some RNA modifications are directly involved in the regulation of these processes. RNA epigenetics is emerging as a mechanism to achieve dynamic regulation of RNA function. Other modifications may prevent or be a signal for degradation. All types of RNA species are subject to processing or degradation, and numerous cellular mechanisms are involved. Unexpectedly, several studies during the last decade have established a connection between DNA and RNA surveillance mechanisms in eukaryotes. Several proteins that respond to DNA damage, either to process or to signal the presence of damaged DNA, have been shown to participate in RNA quality control, turnover or processing. Some enzymes that repair DNA damage may also process modified RNA substrates. In this review, we give an overview of the DNA repair proteins that function in RNA metabolism. We also discuss the roles of two base excision repair enzymes, SMUG1 and APE1, in RNA quality control.

## Introduction

Cellular RNA is susceptible to chemical modification and so far, 66 different modifications have been identified in eukaryotes [The RNA Modification Database (RNAMDB, http://mods.rna.albany.edu/)]. Some of these modified bases have well known regulatory functions; pseudouridine is, for example, the most common post-transcriptionally introduced RNA modification, and is particularly important for structure and function of ribosomal RNA (rRNA) and small nuclear RNA (snRNA) [1]. Pseudouridine is introduced enzymatically by pseudouridylases that isomerise uridine, and the importance of this modification is illustrated by the premature aging syndrome dyskeratosis congenita, which may result from mutations in the main human pseudouridylase, Dyskerin (DKC1) [2]. Moreover, RNA editing mechanisms rely on enzymatically introduced base modifications to generate codon changes, like the deamination of cytidine to uridine in the apolipoprotein B mRNA by Apolipoprotein B Editing Catalytic subunit 1 (APOBEC1) [3], which generates a premature stop codon [4]. Interestingly, many modified bases found in RNA are lesions when present in DNA: inosine results from deamination of adenosine and is a mild mutagenic lesion in DNA [5], but inosine is introduced enzymatically into RNA by adenosine deaminases as a strategy to create transcriptional diversity. RNA is also subject to spontaneous chemical modification, and due to its localization and single-stranded nature, is even more susceptible to oxidation than DNA [6]. Dedicated DNA repair enzymes correct lesions in DNA using the undamaged strand as a template for restoration of the original sequence. Similar RNA repair mechanisms seem unlikely, due to the lack of a template for accurate repair. The exception would be direct repair strategies that reverse RNA modifications without any need for templated resynthesis. The groundbreaking discovery that ALKBH3, a member of the AlkB family of iron and 2-oxoglutarate-dependent dioxygenases, has demethylation activity on RNA substrates containing 1-methyladenosine (m^1^A) and 3-methylcytidine (m^3^C) raised the exciting hypothesis that RNA modifications may be enzymatically repaired or reversed in mammalian cells [7]. Regulated enzymatic introduction of RNA modifications for which there exists an enzymatic reversal strategy is analogous to DNA epigenetic regulation. Indeed, RNA epigenetics has recently been described as a mechanism to achieve dynamic regulation of gene expression [8]. Apart from their implication in many cellular processes, RNA modifications may also prevent or be a signal for degradation. A convergence of DNA and RNA metabolism is emerging that may have implications for our understanding of RNA quality control mechanisms. Here, we will give an overview of the main RNA quality control mechanisms and discuss recent developments implicating Base Excision Repair (BER) proteins in RNA quality control mechanisms.

## RNA quality control mechanisms

**Fig. 1:**
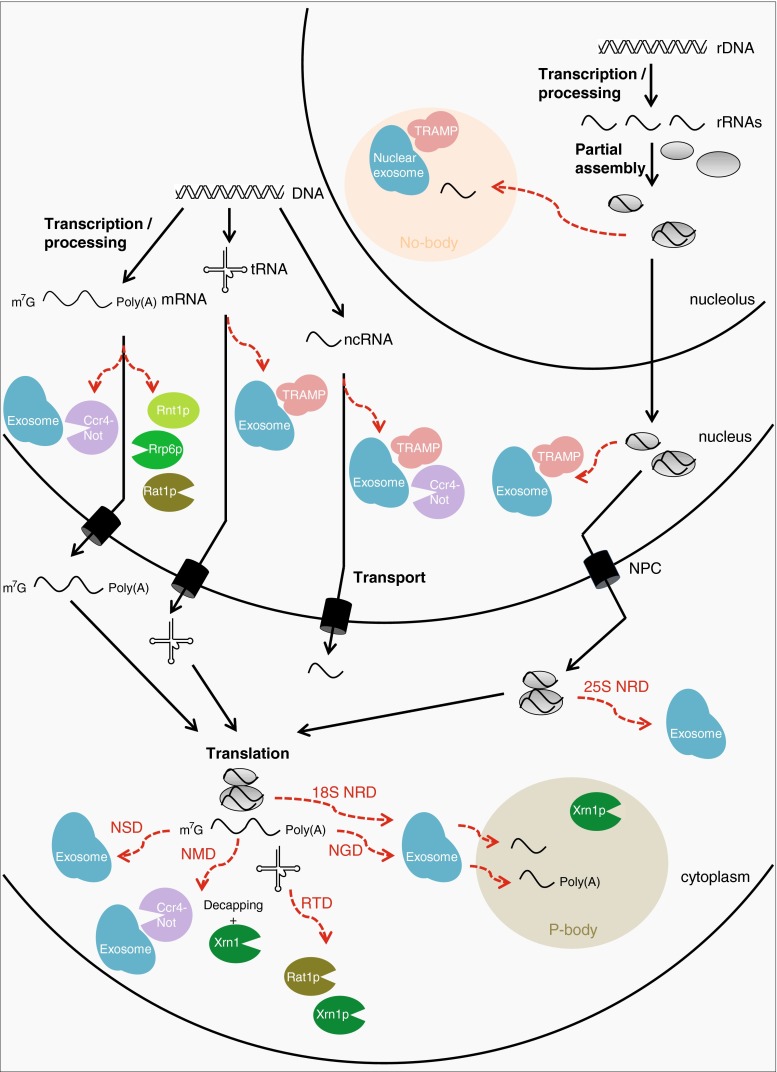
Cellular localisation and RNA targets of the different RNA quality control complexes. The figure depicts some of the known RNA quality control systems for aberrant mRNA, tRNA, rRNA and other non-coding RNA (ncRNA) species in eukaryotic cells. These and additional quality control mechanisms are described in the Text

Eukaryotes possess numerous mechanisms that process or eliminate specific RNA molecules including rRNAs, tRNAs and mRNAs (Fig. 1). Superficially, there are two major modes of RNA degradation; via specialised RNA nucleases (endonucleases or 5′–3′ exonucleases) or via the exosome. The exosome is an RNA degradation factory present in both the nucleus and the cytoplasm that has a central function in RNA metabolism. It is responsible for degradation, processing and regulated turnover of all classes of RNA in eukaryotes [9]. The exosome is a multiprotein complex that structurally resembles the proteasome, with a barrel-shaped central channel into which the substrate is funnelled. In contrast to the proteasome, where the catalytic activity is provided within the core complex, the core exosome is catalytically inactive and instead appears to serve as a substrate-binding scaffold onto which individual nucleases assemble [10]. The nuclear exosome is endowed with three different ribonuclease activities; endonuclease and 3′–5′ exonuclease activities are provided by the Rrp44 subunit and a second 3′–5′ exonuclease activity is contributed by the Rrp6 subunit [11].

The exosome degrades RNA to single-nucleotide monophosphates and a residual four-nucleotide fragment, but it can only degrade RNA substrates that enter the core with their 3′ ends [12]. This means that the substrate cannot enter in a fully folded state and therefore, that the exosome, like the proteasome, requires accessory and regulatory factors to prepare the substrate for degradation. Degradation by the exosome requires the generation of an exposed 3′-hydroxyl (3′-OH) single-stranded RNA end that can enter the exosome core and serve as a substrate for its exonucleolytic activity. The exosome may be the final destination in the process of degradation for all classes of RNA, but the preparatory steps vary depending on the type and location of the substrates. However, there are degradation strategies that may act entirely independent of the exosome and sometimes in collaboration with the exosome (Fig. 1).

Cytoplasmic mRNAs subject to degradation are either deadenylated prior to 3′ to 5′ degradation by the cytoplasmic exosome [13], or decapped and then degraded by the 5′–3′ exonuclease Xrn1 [14, 15]. Deadenylation involves the collaboration between one of the two deadenylases Ccr4 and Caf1 of the Ccr4-Not complex and the related deadenylase Pan2/3 [16]. The removal of the poly(A) tail is followed by degradation by the exosome. Aberrancies in translation are processed by specialised mRNA decay pathways; the non-stop decay (NSD) detects and degrades mRNAs lacking a stop codon [17] and the no-go decay (NGD) targets mRNAs bound by ribosomes that are stalled in translation elongation [18], whereas the nonsense-mediated decay (NMD) promotes degradation of mRNAs undergoing premature translation termination [19].

Nuclear mRNAs may also be degraded when processing or export is altered. In these cases, mRNAs are degraded by the nuclear exosome [20], or, at least in yeast, cleaved by the endonuclease Rnt1p and then degraded by the nucleases Rrp6p and Rat1p [21]. A recent study revealed that the Ccr4-Not complex may be required to tether misprocessed mRNAs to sites of transcription to prevent their export or to act as a scaffold to recruit the exosome to degrade them [22]. In mammals, a deadenylation-dependent decay pathway targeting aberrant nuclear mRNAs has been identified [23], but none of the nucleases have been characterised thus far. The recent discovery that unprocessed nuclear mRNAs remain tethered to the DNA template in association with RNA polymerase II in an Rrp6-dependent manner suggests that the exosome is required for degradation of those aberrant RNAs in mammals [24].

The functional noncoding rRNAs and tRNAs may also be degraded both in the cytoplasm and the nucleus. Upon translation failure, cytoplasmic rRNAs are degraded by a process referred to as nonfunctional ribosomal decay (NRD) [25]. Introduction of deleterious mutations, in either the 25S peptidyl transferase center or 18S decoding site, lead to the downregulation of those rRNAs, via decreased stability of the mature rRNA found in fully assembled ribosomes [26]. 25S NRD substrates, which accumulate around the nuclear envelope, are eliminated after export to the cytoplasm in a process involving the exosome [26]. 18S NRD is, on the other hand, dependent on translation elongation and utilises the same proteins as those participating in NGD. In both cases, the stalled translation complexes are processed by the exosome and then further degraded by Xrn1 in cytoplasmic processing bodies (P-bodies) [26]. The lack of different combinations of modifications in mature tRNA molecules leads to their degradation via the rapid tRNA degradation (RTD) pathway [27]. The RTD pathway involves the 5′–3′ exonucleases Rat1 and Xrn1 [28].

In the nucleus, tRNAs and rRNAs having maturation defects can be polyadenylated by the Trf4-Air2-Mtr4 polyadenylation (TRAMP) complex before degradation by the nuclear exosome. The TRAMP complex adds short poly(A) tails to aberrant transcripts, forming a favourable substrate for the exosome and thus facilitating RNA digestion by the exosome [29]. In addition, non-coding nuclear snRNAs and snoRNAs, whose turnover and/or processing need the nuclear degradation machinery, are also affected by the Ccr4-Not complex, suggesting that Ccr4-Not connects TRAMP with the nuclear exosome for processing and/or degradation of their target RNAs [30, 31]. Further investigation of the quality control mechanisms that detect and degrade irregular pre-rRNAs showed that they are degraded within a sub-nucleolar structure termed the No-body [32]. Pre-ribosome components, polyadenylated RNAs, TRAMP and the exosome accumulate in the No-body, in which pre-ribosome surveillance is likely to take place. Other nuclear pre-rRNA surveillance pathways have been described in *Saccharomyces cerevisiae*; in the absence of pre-rRNA dimethylation, for example, Dim1p blocks pre-rRNA processing steps required for maturation of 18S rRNA [33].

## Regulation of RNA metabolism

### RNA turnover

Degradation of mRNAs plays an important role in regulating the level of mRNA transcripts. This is the case of the c-myc mRNA, which is degraded via two distinct pathways. One pathway comprises deadenylation followed by 3′–5′ exonucleolytic degradation [34]. Another pathway, which also applies for other mRNAs, involves endonucleolytic cleavage [35].

Endoribonucleolytic RNA degradation may be used as a strategy for cells to respond quickly to external stimuli. For example, tRNAs and rRNAs have been shown to be cleaved by the yeast endonuclease Rny1 upon oxidative stress [36]. This does not appear to be a mechanism for quality control, as misprocessed RNAs do not increase in RNA processing mutants. Instead, tRNA cleavage could contribute to translational repression by reducing the functional levels of tRNA during cellular stress [36]. Lately, RNA methylation by Dnmt2 was shown to protect tRNAs against stress-induced cleavage [37], which supports that specific modifications may affect tRNA turnover. Moreover, large amounts of tRNA precursors are degraded by the exosome even in the absence of processing defects and stress [38], thereby revealing a major pathway of pre-tRNA turnover that competes with tRNA maturation. tRNA cleavage upon stress was also suggested to have a broader cellular function, since the cleaved or nicked tRNAs might inhibit mRNA function through several potential mechanisms [39].

### RNA maturation

As part of the RNA maturation process, specific cleavages and modifications of RNA molecules are essential for producing stable mRNAs and functional non-coding RNAs. Splicing of tRNAs involves the precise removal of intronic sequences, which requires two incisions of the pre-tRNA at the exon–intron borders. The cleavage reactions are catalysed by a tRNA splicing endonuclease that recognises the structure of the pre-tRNA and yields 2′–3′-cyclic phosphate and 5′-OH termini in the cleaved tRNA and the excised linear intron [40]. In eukaryotes, correct splicing of the pre-tRNA requires a highly conserved adenosine: inosine (A:I) base pair [41], which illustrates the importance of RNA modifications for proper processing. In eukaryotes, three of the four rRNAs (18S, 5.8S and 28S) are synthesised from a single pre-rRNA molecule by RNA polymerase I. Maturation of rRNA includes specific cleavage steps and involves nucleolar proteins, snoRNAs, putative RNA helicases, and a number of nucleases. Rnt1 shows endonuclease activity both on 5′ and 3′ external transcribed spacers (ETS) [42]. The RNase MRP also shows specific endoribonucleolytic activity on pre-rRNA in vitro [43]. Moreover, the 5.8S, 18S and 25S rRNA species are efficiently processed by the Rrp43p subunit of the exosome [44], indicating that the exosome is required for maturation of those rRNA species.

Pre-RNAs are not only cleaved to generate mature RNA molecules, but are also modified at specific bases. Pseudouridylation has thus far been considered to provide constitutive modifications. Recently, it was demonstrated that pseudouridylation in U2 snRNA can be conditionally induced in *Saccharomyces cerevisiae* with impact on pre-mRNA splicing [45]. This interesting finding raises the exciting hypothesis that inducible RNA modifications may be a general mechanism of fine-tuning cellular responses, and importantly, that they may be reversible. Enzyme-catalysed modification of the wobble nucleosides in tRNA affects anticodon positioning in the ribosome. The yeast tRNA methyltransferase 9 (Trm9) catalyses the formation of 2,5-methoxycarbonylmethyluridine (mcm^5^U) and 5-methoxycarbonylmethyl-2-thiouridine (mcm^5^s^2^U), classified as key determinants of translational fidelity [46]. In mammals, several tRNAs have 5-methoxycarbonylmethyluridine (mcm^5^U), or derivatives thereof, in the wobble position, which are believed to restrict wobbling and improve translational efficiency. Recently, the ALKBH8 member of the AlkB protein family was reported to methylate 5-carboxymethyluridine (cm^5^U) and 5-carboxymethyl-2-thiouridine (cm^5^s^2^U) and, thus, participate in the maturation of the tRNA^Sec^, tRNA^Glu^ and tRNA^Arg^ [47, 48]. It is interesting to note that AlkB proteins were initially characterised for their ability to remove alkylation damage in DNA [49]. An additional hydroxylation activity was recently found for ALKBH8 on tRNA^Gly^, further expanding the function of the ALKBH oxygenases beyond nucleic acid repair [50].

### RNA epigenetics

The concept of RNA epigenetics emerged with the discovery that post-transcriptional RNA modifications can be dynamic and might have regulatory roles analogous to those of epigenetic DNA modifications [8]. One example is *N* ^6^-methyladenosine (m^6^A), which is one of the most common modifications in RNA. It is introduced enzymatically by an unidentified methyltransferase complex containing the subunit methyltransferase-like 3 (METTL3) [51], and erased by the fat mass and obesity-associated (FTO) enzyme [52]. FTO performs direct demethylation of the adenine base using its oxidative demethylation activity and partially localises with nuclear speckles [53]. Similarly to FTO, ALKBH5 was recently shown to demethylate m^6^A in mRNA [54]. This demethylation activity of ALKBH5 significantly affects mRNA export and assembly of mRNA processing factors in nuclear speckles [54]. Moreover, *Alkbh5*-deficient male mice have increased m^6^A in mRNA and are characterised by impaired fertility [54], demonstrating that the reversible m^6^A modification has fundamental and broad functions in mammalian cells. RNA epigenetics may thus represent an example of how DNA repair enzymes, or DNA repair-like enzymes, may contribute important functions beyond the regulation of RNA metabolism.

### RNA editing

RNA editing changes the coding properties of an RNA molecule relative to that of the encoding DNA. Substitutions and insertions/deletions are the two types of editing occurring in all types of cellular RNA. Adenosine-to-inosine (A-to-I) RNA editing is a central generator of transcriptome diversity and regulation in eukaryotes. The reaction is catalysed by a family of adenosine deaminases acting on RNA (ADARs). A-to-I editing of the tRNA anti-codon plays a crucial role in the function of tRNAs during protein synthesis and is performed by enzymes homologous to the ADAR; adenosine deaminase acting on tRNA (ADATs) [55]. Recently, human endonuclease V (ENDOV) was reported to cleave I-containing RNA oligonucleotides corresponding to a part of the Gabra-3 transcript, a known substrate for ADAR1 and ADAR2 [56], and oligonucleotides corresponding to the anti-codon loop in tRNA^Arg^ [57]. These findings may suggest that ENDOV could antagonise the effect of adenosine deaminase enzymes by destruction of I-containing RNAs, or that it may act together with ADAR to eliminate A:I-containing transcripts.

*Escherichia coli* EndoV is, in contrast, a DNA repair enzyme that recognises a wide range of modified bases and cleaves the DNA in addition to I-containing RNA [57, 58]. Interestingly, *E. coli* has no ADAR enzyme but has an ADAT enzyme, and therefore likely utilises inosine as a modified base only in the tRNA wobble position. In higher eukaryotes that express ADAR enzymes, inosine may have become a more widely used regulatory modification in RNA.

## Convergence between DNA and RNA surveillance pathways

Recent data indicate that some proteins responding to DNA damage may function in RNA metabolism. The DNA repair enzyme 5′-tyrosyl-DNA phopshodiesterase-2 (TDP2) cleaves the protein-RNA linkage generated by picornaviruses, and thus has a VPg unlinkase activity, a unique RNA repair-like function [59]. TDP2 represents one example of an increasing number of DNA repair enzymes that have demonstrated or predicted functions in RNA metabolism (Table 1). Moreover, many proteins involved in the signalling of DNA damage also function in RNA metabolism; the two components Rtt101 and Mms1 of an E3 ubiquitin ligase complex involved in repair of DNA damage during replication are also required for the 25S NRD [60]. The RUVBL1 and RUVBL2 proteins, which participate in the chromatin remodelling at sites of DNA damage, were reported to function in NMD [61]. Poly (ADP-ribose) polymerase 1 (PARP1), which adds (ADP-ribose) polymers to single-stranded DNA breaks as part of the DNA damage response, has recently been shown to have several functions in RNA biology: PARP1 localises within nucleoli and Cajal bodies and contributes to Cajal body formation, and it has been suggested that PARP1 controls protein trafficking through the Cajal body [62]. PARP1 enzymatic activity is required for targeting nucleolar proteins to the proximity of the precursor rRNA, and thereby controls pre-rRNA processing and pre-ribosome assembly [63] (Table 1). The association of PARP1 with hnRNP A2/B1 and several ribosomal proteins [64] corroborates its implication in ribosomal biogenesis.

**Table 1:** List of DNA repair proteins shown to be linked with RNA metabolism

Name	Role in DNA repair	Localisation in compartments connected to RNA metabolism^a^	Interaction partners connected to RNA metabolism	RNA association/activity on RNA	References
APE1	AP endonuclease	Nucleolus	Nucleophosmin NPM1	Association with 47S, 28S and 18S rRNAs and endonuclease activity	[95]
SMUG1	Uracil-DNA glycosylase	Nucleolus, Cajal body	Pseudouridine synthase DKC1	Association with 47S rRNA, activity on in vitro RNA substrates	[107]
OGG1	8-oxoguanine-DNA glycosylase	Nucleolus	ND	ND	[77]
NEIL1	DNA glycosylase	Nucleolus	ND	ND	[78]
ENDOV	Endonuclease	Nucleolus	ND	Incision activity on tRNA	[57]
ABH3	DNA demethylase	Nucleolus	ND	Activity on RNA homopolymers	[7]
FTO	DNA demethylase	Nuclear speckles	ND	Oxidative demethylation on RNA	[53]
TDP2	DNA phosphodiesterase	ND	ND	Unlinkase activity on RNA	[59]
PARP1	Poly(ADP-ribose) polymerase	Nucleolus, Cajal body	hnRNP A2/B1 RPL22, RPL30, RPS4, RPL23a, RPS6, RPL18a, RPL14, RPL21, RPS13	ND	[62–64]
FEN1	Flap endonuclease	Nucleolus	hnRNP A1	ND	[72, 114]

In terms of cellular localisation, interaction partners and/or activity on RNA

^a^Genes with Gene Ontology annotation Base Excision Repair (GO:0006284) with nucleolar localization (GO:0005730) were extracted from The Gene Ontology Website (http://www.geneontology.org/), and the intersection identified

Conversely, RNA processing proteins or complexes may be involved in the DNA damage response or exhibit DNA repair activities. For instance, the Ccr4-Not complex promotes transcription coupled nucleotide excision repair [65]. The very high rates of mutagenesis and transcription-dependent recombination of DNA in RNA-processing mutants show that RNA processing enzymes contribute to genomic stability [66]. The Trm9 enzyme modifying the uridine wobble base in specific tRNAs was identified as a potential promotor of the DNA damage response; a Trm9Δ allele increases cell sensitivity to methyl methanesulfonate (MMS) and γ-irradiation [67–69]. The *Drosophila* ribosomal proteins S3 and PO exhibit apurinic/apyrimidinic (AP) endonuclease activities in vitro [70, 71]. The heterogeneous nuclear ribonucleoprotein A1 (hnRNP A1), initially shown to influence pre-mRNA processing, was reported to interact with and stimulate the activity of the flap endonuclease 1 (FEN1) participating in DNA replication and repair [72] (Table 1). Loss of the mRNA splicing factor SC35 results in genomic instability [73] and the serine-arginine-rich (SR) protein ASF/SF2 that regulates the early steps of splicing acts to prevent transcription-associated genomic instability [74]. These two last examples illustrate the role of RNA processing enzymes in the maintenance of genomic integrity and how the different DNA and RNA processing pathways are integrated and interconnected in the eukaryotic nucleus.

## Base excision repair proteins in RNA metabolism

**Fig. 2:**
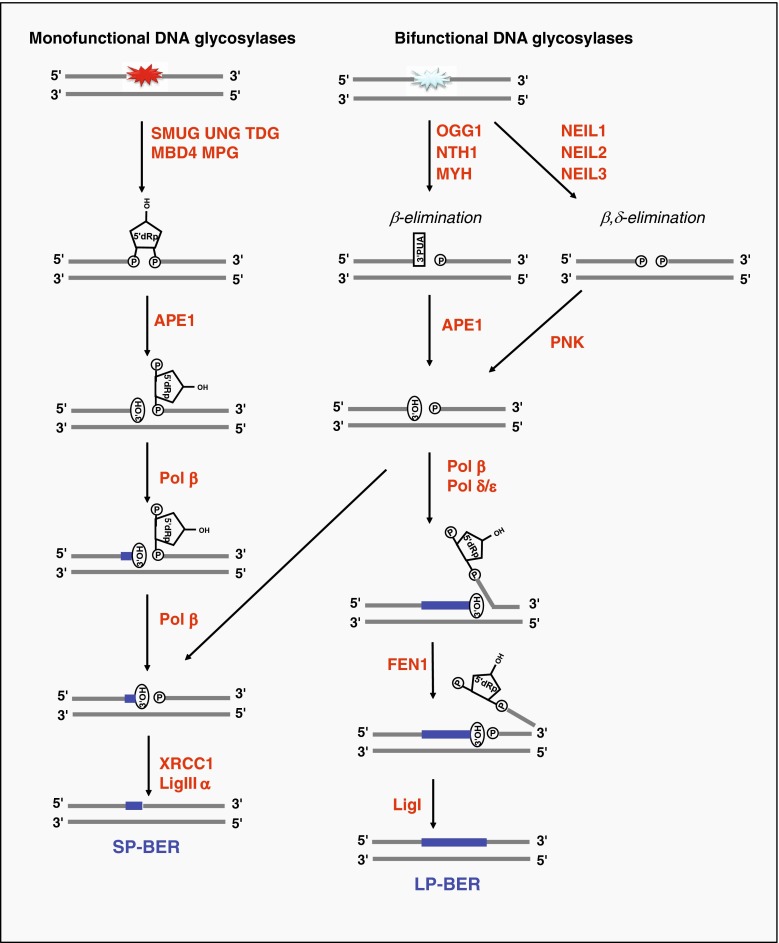
The base excision repair (BER) pathway. Monofunctional DNA glycosylases (UNG, SMUG1, TDG, MPG, MBD4) excise the damaged base leaving an abasic site (AP site) in DNA. Subsequently, the DNA backbone is incised 5′ to the AP site by AP-endonuclease 1 (APE1), which directly generates a strand break with 3′-hydroxyl (3′-OH) group and a 5′-deoxyribose-5-phosphate (5′-dRP) end. Bi-functional DNA glycosylases (NEIL1/2/3, OGG1, NTH1, MYH) utilise an amino group of the enzyme rather than a water molecule as a nucleophile to cleave the *N*-glycosidic bond. Resolution of the resulting Schiff-base intermediate in a β-elimination reaction leads to incision of the DNA backbone 3′ to the AP site. This is referred to as the AP lyase activity and results in a single strand break with 3′-phospho-α,β-unsaturated aldehyde (3′-PUA) and 5′-phosphate (5′-P) ends. APE1 generates 3′-OH termini using its 3′-phosphodiesterase activity. NEIL1 and NEIL2 utilise a β,δ-elimination reaction with removal of the deoxyribose residue and generation of 3′-phosphate termini. The 3′-phosphate may be removed by polynucleotide kinase (PNK) in an APE1-independent BER sub-pathway. BER proceeds further via two alternative sub-pathways: short-patch (SP) repair, which involves replacement of one nucleotide, or long-patch (LP) repair, which involves replacement of several nucleotides (at least two, and often 6–13 nucleotides). In SP-BER, DNA polymerase β (Pol β) inserts a new nucleotide and removes the 5′-dRP moity using an intrinsic lyase activity, before the nick is sealed by XRCC1 and DNA ligase III (LigIII). If, however, modification of the 5′-end is modified in some way that prevents its excision by Pol β, BER will be funnelled into the LP-BER pathway, which utilises DNA replication factors and synthesises a longer stretch of DNA (2–13 nucleotide-long repair patches have been reported). The 5′-terminal moiety is then removed as part of an oligonucleotide by Flap endonuclease (FEN1). The resulting nick is sealed by DNA ligase I (LigI)

Several lines of evidence implicate enzymes that primarily function in the BER pathway in various aspects of RNA metabolism. BER is a well-conserved pathway where damaged or modified bases are excised and replaced through a series of coordinated steps [75]. BER is initiated by a DNA glycosylase that senses chemical modifications of DNA bases [76]. In mammalian cells, there are 11 different DNA glycosylases with overlapping substrate specificities that together have the ability to remove a wide range of damaged bases resulting from spontaneous or enzymatic hydrolysis, oxidation, and alkylation reactions. DNA glycosylases initiate repair by hydrolysing the *N*-glycosidic bond linking the base and the DNA backbone. DNA glycosylases are generally classified as being either mono-functional or bi-functional, with the latter having an associated DNA lyase activity. However, the associated AP lyase activity may not always be used. Following excision of the damaged base, BER can proceed through one of several different routes of the major modes of repair, which are illustrated in Fig. 2; the AP endonuclease APE1 is a multifunctional enzyme with a central function in BER. APE1 introduces a nick in the DNA backbone after removal of the damaged base, but may also clean up and trim DNA ends in order to generate the 3′-OH groups that are substrates for DNA polymerases. BER proceeds further via two alternative sub-pathways: short-patch (SP) repair, which involves replacement of one nucleotide by specialised DNA polymerases, or long-patch (LP) repair, which involves replacement of several nucleotides using the general replication machinery.

Many BER proteins, like the DNA glycosylases OGG1 and NEIL1, accumulate in the nucleolus [77, 78]. The localisation of OGG1 to nucleoli is dynamic and responsive to external stimuli; OGG1 accumulates first in nucleoli and later in cytoplasmic stress granules after heavy metal stress [79]. OGG1 localisation depends on rDNA transcription by RNA polymerase I [77]. Moreover, immunoprecipitation experiments performed in many different laboratories revealed associations between BER proteins and proteins implicated in transcription. For example, YB-1, a multifunctional protein involved in the regulation of transcription, translation, and mRNA splicing, interacts with and regulates the activity of APE1 [80], as well as the NTH1 [81] and NEIL2 [82] DNA glycosylases. However, for most of these proteins, the current evidence suggests that the biological significance of these interactions and associations may be to promote DNA repair in the rDNA locus. There are two exceptions, APE1 and SMUG1, where nucleolar localisation is accompanied by a demonstrated function in RNA metabolism; these will be discussed below.

### APE1, a multifunctional enzyme in DNA and RNA metabolism

APE1 (also known as Hap1, Apex or Ref1, APN1) is a multifunctional protein. It was first shown to stimulate the DNA-binding activity of the proto-oncogenes c-fos and c-jun by a reduction–oxidation (redox) mechanism [83]. Later, APE1 was reported to have a role in transcription regulation since it potentiates the binding of specific DNA elements to several transcription factors through alterations of their redox state [84]. Further investigation showed that APE1 possesses transcription repressor activity upon a rise in extracellular calcium [85]. The repair activity of APE1 was discovered in bovine cells, with the purification of the major factor for repair of bleomycin-induced DNA strand breaks [86]. APE1 has a broad specificity for AP sites and incises them to generate a 3′-OH and a 5′-terminal deoxyribose-5-phosphate (5′-dRP) residue [87]. In addition to its AP endonuclease activity, APE1 possesses phosphodiesterase [88] and phosphatase [89] activities, as well as a weak 3′-exonuclease activity for duplex DNA [87].

Ape1 is essential for early embryonic development in mice, since deletion of both alleles of *Ape1* leads to early lethality [90]. However, it is not yet known which activity of Ape1 is required for mouse viability. APE1 has been reported to influence many different biological processes. APE1 has a major repair role in mitochondria in which mitochondrial DNA is highly susceptible to oxidative damage [91].

Moreover, APE1 interferes with the granzyme A-induced cell death response [92]. Recent findings have highlighted a novel role for APE1 in RNA metabolism. First of all, APE1 exhibits endonuclease activity on single-stranded AP site containing RNA molecules, as well as various lesion-containing DNA/RNA hybrids [93]. Moreover, the APE1 endonuclease activity is involved in the regulation of mRNA turnover. APE1 downregulates c-myc expression in vivo by preferentially cleaving between UA and CA di-nucleotides located in the coding region determinant of c-myc mRNA [94]. Knockdown of APE1 in HeLa cells leads to an increase in c-myc mRNA expression, suggesting that APE1 endonuclease activity on c-myc controls its abundance [94].

In an attempt to identify APE1 interaction partners, the nucleophosmin 1 (NPM1) protein was found to directly interact with the N-terminal region of APE1 [95]. Interestingly, the disordered APE1 N-terminal is a recent evolutionary acquisition and may thus represent a “gain of function” [96]. Both NPM1 and APE1 localise within nucleoli during S phase [95]. The interaction with NPM1 stimulates APE1 endonuclease activity on abasic DNA, while it inhibits the incision activity on abasic RNA. Moreover, APE1 associates in vivo with 47S, 28S and 18S rRNA species, regulates rRNA oxidation levels, and its interaction with NPM1 is disrupted upon oxidative stress. These findings suggest that NPM1 exerts a fine-tuning control of APE1 endonuclease activity within nucleoli to promote repair of AP sites in rDNA and remove oxidised rRNA molecules. Moreover, the APE1/NPM1 association is impaired during oxidative stress [95], suggesting that the protein may be released from the nucleolus during stress conditions. In accordance with these observations, a working model was proposed in which APE1 activity is mainly focused on rRNA quality control in the nucleolus, whereas during the DNA damage response, the re-localisation of APE1 and NPM1 into the nucleoplasm leads to the activation of the APE1 DNA repair function [97]. Hence, APE1 is a BER enzyme that functions in the rRNA cleansing process, to maintain a functional RNome and to regulate mRNA expression through mRNA decay.

### SMUG1 contributes to RNA quality control

SMUG1 was identified in a genome-wide screen for DNA glycosylases on the basis of its ability to bind synthetic inhibitors [98]. SMUG1 belongs to the uracil-DNA glycosylase (UDG) superfamily, which also includes the UNG, MBD4, and TDG enzymes. SMUG1 removes uracil in single-stranded and double-stranded DNA efficiently and has high affinity for binding the product, the AP site [99].

The crystal structure of SMUG1 from *Xenopus laevis* bound to double-stranded DNA substrates confirmed the conservation of the core structural fold common to the UDG superfamily, and that the enzyme utilises the characteristic mechanism of extrahelical pyrimidine recognition [100]. Further functional analysis of a series of mutants of human SMUG1, in conjunction with homology modelling of the human SMUG1 structure, revealed that the Asn85 and His239 residues are crucial for the hydrolysis of the *N*-glycosidic bond, Phe98 and Asn163 for the discrimination of pyrimidine rings, and Gly87 and Met91 for the recognition of the C5 substituent [101].

In addition to the activity on uracil substrates, SMUG1 has a special function to repair oxidised pyrimidines; it excises uracil derivatives bearing an oxidised group at the C5 position, such as 5-hydroxyuracil (hoU), 5-hydroxymethyluracil (hmU), and 5-formyluracil (fU), but not the analogous cytosine derivatives 5-hydroxycytosine (hoC) and 5-formylcytosine (fC) [102]. Recently, an excision activity of human SMUG1 of the deaminated base xanthine from single-stranded and double-stranded DNA was also described [103]. *Smug1* inactivation resulted in loss of all detectable hmU-excision activity, indicating that Smug1 is the major, if not the only, enzyme responsible for hmU excision in the mouse [104].

SMUG1 is a recent evolutionary acquisition; it is conserved among coelomata but absent in nematodes, plants, yeast, and bacteria. SMUG1 was originally suggested to have evolved in higher eukaryotes as an anti-mutator distinct from the UNG enzyme [105, 106]. Consistently, a ten-fold increase in spontaneous C:G to T:A transitions was observed in cells deficient in Smug1 [106]. However, although loss of Smug1 increased the cancer predisposition of mice lacking the mismatch repair enzyme Msh2 [104], neither mice harbouring a targeted inactivation of *Smug1* nor *Ung/Smug1* double-deficient mice show any obvious phenotype [104].

Recently, we showed that SMUG1 directly interacts with the pseudouridine synthase DKC1 [107]. DKC1 catalyses the pseudouridylation of specific uridine-residues in rRNA, which is essential for the functionality of those RNA molecules. Mutagenesis of SMUG1 identified three amino acids essential for DKC1 binding, including the glutamic acid residues at position 29 and 33. These two residues are part of the unstructured and flexible nonconserved amino-terminal domain of the protein [105]. It remains to be demonstrated whether this particular region of SMUG1 may account for its putative recently acquired non-canonical activity in RNA metabolism similar to the unstructured amino-terminal domain of APE1 required for NPM1 interaction. Both SMUG1 and DKC1 localise within nucleoli and Cajal bodies, in which rRNA biogenesis and non-coding RNA maturation, respectively, take place. Most importantly, SMUG1 associates in vivo with the 47S precursor rRNA. DKC1 was suggested to participate in rRNA quality control by mediating the degradation of damaged rRNA by the nuclear exosome [108]. Thus, we tested whether depletion of SMUG1 affected rRNA biogenesis. We found that SMUG1 depletion was accompanied by a reduction of the expression levels of the three mature 28S, 18S and 5.8S rRNA species. The reduced expression of mature rRNA species was accompanied by an increase in rRNA polyadenylation, indicating that SMUG1 depletion led to accumulation of aberrant rRNA species that is targeted for degradation. Hence, these results suggest that the BER enzyme SMUG1 participates in rRNA quality control [107]. It is currently unclear exactly how SMUG1 affects rRNA biogenesis, but we found that SMUG1 has incision activity on hmU-containing RNA substrates in vitro, and that there is an increased hmU-content in 28S and 18S rRNAs isolated from SMUG1-depleted cells [107]. The hmU-modification was not previously demonstrated to be a natural modification of RNA (http://mods.rna.albany.edu/), yet our data show that it is present in human rRNA in the absence of exogenous stress. It might originate from incorporation of hmU recycled from damaged DNA, as previously suggested [109]. However, hmU may also result from hydrolytic deamination of 5-hydroxymethylcytosine, which is a natural modification found in 18S and 28S rRNAs in eukaryotes [110].

### How can BER enzymes contribute to the RNA quality control process?

A genetic interaction between the exosome subunit *RRP6* and the BER pathway was reported in *Saccharomyces cerevisiae* [111]. *Rrp6* and *apn1* (AP endonuclease) single mutants showed similar growth rates, as compared with the double mutant strain, upon 5-fluorouracil (5FU) treatment, which induces both DNA and RNA damage [111]. This epistatic effect indicated that Rrp6p and APN1 act in the same pathway. The biological significance of this interaction was difficult to interpret, since mutation in the *UNG1* gene did not rescue the growth of both single mutants [111], as would be expected according to the BER activity of these enzymes (Fig. 2). However, the mismatch repair pathway was recently shown to act upstream of APN-1 in the 5FU response in *Caenorhabditis elegans* [112]. Therefore, the genetic interaction between *APN1* and *RRP6* may be interesting, and in fact be the first indication of a connection between DNA repair and RNA surveillance pathways.

In contrast to the deep understanding of the RNA degradation processes of RNA quality control, the mechanisms that control the specificity of RNA degradation are still poorly defined. Many different types of aberrations may trigger RNA degradation, and it is unlikely that a single quality control system is capable of precisely recognising such diverse aberrations. A biochemical rationale for the involvement of BER proteins in RNA quality control could be that the ability of these enzymes to recognise subtle chemical modifications could contribute to identify substrates destined for degradation. Our recent findings demonstrating that the BER enzyme SMUG1 excises RNA-containing hmU and controls hmU levels in 28S and 18S rRNA species, as well as their expression levels [107], are consistent with the latter model. Indeed, hmU may be excised by SMUG1 to initiate specific degradation. If, then, APE1 processes the AP site generated by SMUG1, this will create a 3′-OH terminus. 3′ ends generated by the concerted action of SMUG1 and APE1 may then be targeted by Ccr4-Not, TRAMP or the exosome, for further destruction. Thus, we speculate that SMUG1 and APE1 would act upstream of the degradation machinery to target specific RNA molecules and make them recognisable by the degradation machinery. In this working model, the BER DNA glycosylases, AP endonucleases, and end-processing enzymes (Fig. 2), but not the BER pathway as such, might function to recognise specific modifications in RNA to prepare them for degradation. One may speculate that other BER enzymes having activity on RNA molecules may similarly regulate the fate of their RNA targets.

## Conclusions and perspectives

Because little is known about how aberrant RNA species are recognised and specifically targeted for destruction, one important future challenge is to understand how the quality control machinery distinguishes its RNA substrates from other RNA molecules. As discussed above, BER enzymes may represent one pathway for targeting of specific RNA molecules for degradation. However, other enzymes probably have similar roles, and their identification is crucial to unravel the complexity of RNA quality control mechanisms.

**Fig. 3:**
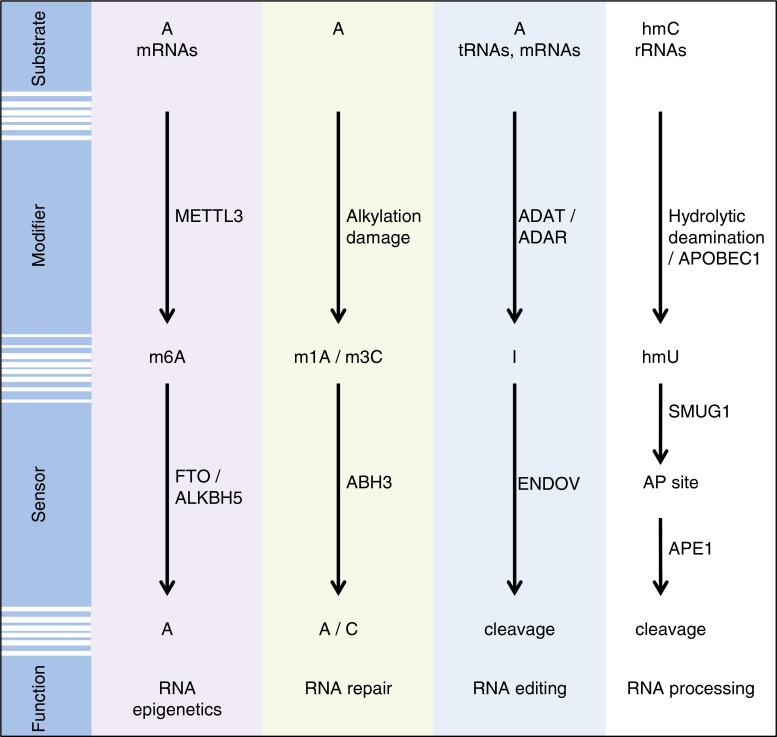
Proposed noncanonical functions of enzymes acting on specific reversible RNA modifications. The *colored boxes* depict demonstrated examples, whereas the *colorless box* describes a hypothetical model of the role of SMUG1 in the processing of hmU in RNA. See Text for more details

There is increasing evidence that RNA modifications may be reversible. One example is m^6^A, which is reversed by FTO by direct demethylation [8, 53] (Fig. 3). Another member of the AlkB family, ALKBH3, was shown to repair alkylation damage in RNA (Fig. 3). Furthermore, ENDOV is thought to regulate A-to-I RNA editing by antagonising the effect of the ADAR enzymes by specific cleavage and destruction of edited transcripts (Fig. 3). A similar function may also be envisioned for SMUG1 on hmU-containing RNA substrates (Fig. 3). 5-hydroxymethylcytosine is a natural modification in 18S and 28S rRNAs in eukaryotes [110], and hmU may therefore result from either hydrolytic deamination of 5-hydroxymethylcytosine or active deamination by APOBEC1 [113]. hmU may then be processed by SMUG1 to generate an AP site and further cleaved by APE1, as part of a specific RNA processing pathway.

In summary, the currently available data point to an emerging role of specific BER repair enzymes in RNA metabolism and RNA surveillance pathways. The unique ability of BER DNA glycosylases to recognise even subtle chemical modifications of nucleic acid bases may serve to distinguish normal and aberrant RNA molecules. The concerted action between DNA glycosylases and APE1 would, in turn, cleave modified RNA molecules. Further research is, however, required to determine whether this may be a general strategy to identify and prepare aberrant RNA molecules for degradation as part of a bona fide RNA quality control pathway. The recently described RNA processing activities of DNA repair enzymes warrant studies into whether their RNA processing functions contribute to pathologies associated with RNA processing defects.

## References

[1] Hamma T, Ferre-D’Amare AR (2006). Pseudouridine synthases. Chem Biol.

[2] Dokal I (2011) Dyskeratosis congenita. Hematology/the Education Program of the American Society of Hematology American Society of Hematology Education Program, pp 480–486. doi:10.1182/asheducation-2011.1.480 10.1182/asheducation-2011.1.480 22160078

[3] Teng B, Burant CF, Davidson NO (1993). Molecular cloning of an apolipoprotein B messenger RNA editing protein. Science.

[4] Chester A, Somasekaram A, Tzimina M, Jarmuz A, Gisbourne J, O’Keefe R, Scott J, Navaratnam N (2003). The apolipoprotein B mRNA editing complex performs a multifunctional cycle and suppresses nonsense-mediated decay. EMBO J.

[5] Lindahl T (1993). Instability and decay of the primary structure of DNA. Nature.

[6] Li Z, Wu J, Deleo CJ (2006). RNA damage and surveillance under oxidative stress. IUBMB Life.

[7] Aas PA, Otterlei M, Falnes PO, Vagbo CB, Skorpen F, Akbari M, Sundheim O, Bjoras M, Slupphaug G, Seeberg E, Krokan HE (2003). Human and bacterial oxidative demethylases repair alkylation damage in both RNA and DNA. Nature.

[8] He C (2010). Grand challenge commentary: RNA epigenetics?. Nat Chem Biol.

[9] Lykke-Andersen S, Brodersen DE, Jensen TH (2009). Origins and activities of the eukaryotic exosome. J Cell Sci.

[10] Makino DL, Halbach F, Conti E (2013). The RNA exosome and proteasome: common principles of degradation control. Nat Rev Cancer.

[11] Chlebowski A, Lubas M, Jensen TH, Dziembowski A (2013). RNA decay machines: the exosome. Biochim Biophys Acta.

[12] Makino DL, Baumgartner M, Conti E (2013). Crystal structure of an RNA-bound 11-subunit eukaryotic exosome complex. Nature.

[13] Anderson JS, Parker RP (1998). The 3′ to 5′ degradation of yeast mRNAs is a general mechanism for mRNA turnover that requires the SKI2 DEVH box protein and 3′ to 5′ exonucleases of the exosome complex. EMBO J.

[14] Muhlrad D, Decker CJ, Parker R (1994). Deadenylation of the unstable mRNA encoded by the yeast MFA2 gene leads to decapping followed by 5′–3′ digestion of the transcript. Genes Dev.

[15] Lejeune F, Li X, Maquat LE (2003). Nonsense-mediated mRNA decay in mammalian cells involves decapping, deadenylating, and exonucleolytic activities. Mol Cell.

[16] Miller JE, Reese JC (2012). Ccr4-Not complex: the control freak of eukaryotic cells. Crit Rev Biochem Mol Biol.

[17] Vasudevan S, Peltz SW, Wilusz CJ (2002). Non-stop decay—a new mRNA surveillance pathway. BioEssays.

[18] Harigaya Y, Parker R (2010). No-go decay: a quality control mechanism for RNA in translation. Wiley Interdiscip Rev RNA.

[19] Kervestin S, Jacobson A (2012). NMD: a multifaceted response to premature translational termination. Nat Rev.

[20] Hilleren P, McCarthy T, Rosbash M, Parker R, Jensen TH (2001). Quality control of mRNA 3′-end processing is linked to the nuclear exosome. Nature.

[21] Danin-Kreiselman M, Lee CY, Chanfreau G (2003). RNAse III-mediated degradation of unspliced pre-mRNAs and lariat introns. Mol Cell.

[22] Assenholt J, Mouaikel J, Saguez C, Rougemaille M, Libri D, Jensen TH (2011). Implication of Ccr4-Not complex function in mRNA quality control in Saccharomyces cerevisiae. RNA (New York, NY).

[23] Conrad NK, Mili S, Marshall EL, Shu MD, Steitz JA (2006). Identification of a rapid mammalian deadenylation-dependent decay pathway and its inhibition by a viral RNA element. Mol Cell.

[24] de Almeida SF, Garcia-Sacristan A, Custodio N, Carmo-Fonseca M (2010). A link between nuclear RNA surveillance, the human exosome and RNA polymerase II transcriptional termination. Nucleic Acids Res.

[25] LaRiviere FJ, Cole SE, Ferullo DJ, Moore MJ (2006). A late-acting quality control process for mature eukaryotic rRNAs. Mol Cell.

[26] Cole SE, LaRiviere FJ, Merrikh CN, Moore MJ (2009). A convergence of rRNA and mRNA quality control pathways revealed by mechanistic analysis of nonfunctional rRNA decay. Mol Cell.

[27] Alexandrov A, Chernyakov I, Gu W, Hiley SL, Hughes TR, Grayhack EJ, Phizicky EM (2006). Rapid tRNA decay can result from lack of nonessential modifications. Mol Cell.

[28] Chernyakov I, Whipple JM, Kotelawala L, Grayhack EJ, Phizicky EM (2008). Degradation of several hypomodified mature tRNA species in Saccharomyces cerevisiae is mediated by Met22 and the 5′–3′ exonucleases Rat1 and Xrn1. Genes Dev.

[29] Vanacova S, Stefl R (2007). The exosome and RNA quality control in the nucleus. EMBO Rep.

[30] Azzouz N, Panasenko OO, Deluen C, Hsieh J, Theiler G, Collart MA (2009). Specific roles for the Ccr4-Not complex subunits in expression of the genome. RNA.

[31] Azzouz N, Panasenko OO, Colau G, Collart MA (2009). The CCR4-NOT complex physically and functionally interacts with TRAMP and the nuclear exosome. PLoS ONE.

[32] Dez C, Houseley J, Tollervey D (2006). Surveillance of nuclear-restricted pre-ribosomes within a subnucleolar region of Saccharomyces cerevisiae. EMBO J.

[33] Lafontaine DL, Preiss T, Tollervey D (1998). Yeast 18S rRNA dimethylase Dim1p: a quality control mechanism in ribosome synthesis?. Mol Cell Biol.

[34] Brewer G (1999). Evidence for a 3′–5′ decay pathway for c-myc mRNA in mammalian cells. J Biol Chem.

[35] Dodson RE, Shapiro DJ (2002). Regulation of pathways of mRNA destabilization and stabilization. Prog Nucleic Acid Res Mol Biol.

[36] Thompson DM, Lu C, Green PJ, Parker R (2008). tRNA cleavage is a conserved response to oxidative stress in eukaryotes. RNA.

[37] Schaefer M, Pollex T, Hanna K, Tuorto F, Meusburger M, Helm M, Lyko F (2010). RNA methylation by Dnmt2 protects transfer RNAs against stress-induced cleavage. Genes Dev.

[38] Gudipati RK, Xu Z, Lebreton A, Seraphin B, Steinmetz LM, Jacquier A, Libri D (2012). Extensive degradation of RNA precursors by the exosome in wild-type cells. Mol Cell.

[39] Thompson DM, Parker R (2009). Stressing out over tRNA cleavage. Cell.

[40] Popow J, Schleiffer A, Martinez J (2012). Diversity and roles of (t)RNA ligases. Cell Mol Life Sci.

[41] Baldi MI, Mattoccia E, Bufardeci E, Fabbri S, Tocchini-Valentini GP (1992). Participation of the intron in the reaction catalyzed by the Xenopus tRNA splicing endonuclease. Science.

[42] Elela SA, Igel H, Ares M (1996). RNase III cleaves eukaryotic preribosomal RNA at a U3 snoRNP-dependent site. Cell.

[43] Lygerou Z, Allmang C, Tollervey D, Seraphin B (1996). Accurate processing of a eukaryotic precursor ribosomal RNA by ribonuclease MRP in vitro. Science.

[44] Zanchin NI, Goldfarb DS (1999). The exosome subunit Rrp43p is required for the efficient maturation of 5.8S, 18S and 25S rRNA. Nucleic Acids Res.

[45] Wu G, Xiao M, Yang C, Yu YT (2011). U2 snRNA is inducibly pseudouridylated at novel sites by Pus7p and snR81 RNP. EMBO J.

[46] Patil A, Dyavaiah M, Joseph F, Rooney JP, Chan CT, Dedon PC, Begley TJ (2012). Increased tRNA modification and gene-specific codon usage regulate cell cycle progression during the DNA damage response. Cell Cycle.

[47] Songe-Moller L, van den Born E, Leihne V, Vagbo CB, Kristoffersen T, Krokan HE, Kirpekar F, Falnes PO, Klungland A (2010). Mammalian ALKBH8 possesses tRNA methyltransferase activity required for the biogenesis of multiple wobble uridine modifications implicated in translational decoding. Mol Cell Biol.

[48] Fu D, Brophy JA, Chan CT, Atmore KA, Begley U, Paules RS, Dedon PC, Begley TJ, Samson LD (2010). Human AlkB homolog ABH8 Is a tRNA methyltransferase required for wobble uridine modification and DNA damage survival. Mol Cell Biol.

[49] Sedgwick B (2004). Repairing DNA-methylation damage. Nat Rev.

[50] van den Born E, Vagbo CB, Songe-Moller L, Leihne V, Lien GF, Leszczynska G, Malkiewicz A, Krokan HE, Kirpekar F, Klungland A, Falnes PO (2011). ALKBH8-mediated formation of a novel diastereomeric pair of wobble nucleosides in mammalian tRNA. Nature Commun.

[51] Bokar JA, Shambaugh ME, Polayes D, Matera AG, Rottman FM (1997). Purification and cDNA cloning of the AdoMet-binding subunit of the human mRNA (N6-adenosine)-methyltransferase. RNA.

[52] Niu Y, Zhao X, Wu YS, Li MM, Wang XJ, Yang YG (2013). N6-methyl-adenosine (m6A) in RNA: an old modification with a novel epigenetic function. Genom Prot Bioinform.

[53] Jia G, Fu Y, Zhao X, Dai Q, Zheng G, Yang Y, Yi C, Lindahl T, Pan T, Yang YG, He C (2011). N6-methyladenosine in nuclear RNA is a major substrate of the obesity-associated FTO. Nat Chem Biol.

[54] Zheng G, Dahl JA, Niu Y, Fedorcsak P, Huang CM, Li CJ, Vagbo CB, Shi Y, Wang WL, Song SH, Lu Z, Bosmans RP, Dai Q, Hao YJ, Yang X, Zhao WM, Tong WM, Wang XJ, Bogdan F, Furu K, Fu Y, Jia G, Zhao X, Liu J, Krokan HE, Klungland A, Yang YG, He C (2013). ALKBH5 is a mammalian RNA demethylase that impacts RNA metabolism and mouse fertility. Mol Cell.

[55] Schaub M, Keller W (2002). RNA editing by adenosine deaminases generates RNA and protein diversity. Biochimie.

[56] Daniel C, Veno MT, Ekdahl Y, Kjems J, Ohman M (2012). A distant cis acting intronic element induces site-selective RNA editing. Nucleic Acids Res.

[57] Vik ES, Nawaz MS, Strom Andersen P, Fladeby C, Bjoras M, Dalhus B, Alseth I (2013). Endonuclease V cleaves at inosines in RNA. Nat Commun.

[58] Rosnes I, Rowe AD, Vik ES, Forstrom RJ, Alseth I, Bjoras M, Dalhus B (2013). Structural basis of DNA loop recognition by endonuclease V. Structure.

[59] Virgen-Slane R, Rozovics JM, Fitzgerald KD, Ngo T, Chou W, Van der Heden van Noort GJ, Filippov DV, Gershon PD, Semler BL (2012). An RNA virus hijacks an incognito function of a DNA repair enzyme. Proc Natl Acad Sci USA.

[60] Fujii K, Kitabatake M, Sakata T, Miyata A, Ohno M (2009). A role for ubiquitin in the clearance of nonfunctional rRNAs. Genes Dev.

[61] Izumi N, Yamashita A, Iwamatsu A, Kurata R, Nakamura H, Saari B, Hirano H, Anderson P, Ohno S (2010). AAA+ proteins RUVBL1 and RUVBL2 coordinate PIKK activity and function in nonsense-mediated mRNA decay. Sci Signal.

[62] Kotova E, Jarnik M, Tulin AV (2009). Poly (ADP-ribose) polymerase 1 is required for protein localization to Cajal body. PLoS Genet.

[63] Boamah EK, Kotova E, Garabedian M, Jarnik M, Tulin AV (2012). Poly(ADP-Ribose) polymerase 1 (PARP-1) regulates ribosomal biogenesis in Drosophila nucleoli. PLoS Genet.

[64] Pinnola A, Naumova N, Shah M, Tulin AV (2007). Nucleosomal core histones mediate dynamic regulation of poly(ADP-ribose) polymerase 1 protein binding to chromatin and induction of its enzymatic activity. J Biol Chem.

[65] Gaillard H, Tous C, Botet J, Gonzalez-Aguilera C, Quintero MJ, Viladevall L, Garcia-Rubio ML, Rodriguez-Gil A, Marin A, Arino J, Revuelta JL, Chavez S, Aguilera A (2009). Genome-wide analysis of factors affecting transcription elongation and DNA repair: a new role for PAF and Ccr4-Not in transcription-coupled repair. PLoS Genet.

[66] Saguez C, Gonzales FA, Schmid M, Boggild A, Latrick CM, Malagon F, Putnam A, Sanderson L, Jankowsky E, Brodersen DE, Jensen TH (2013). Mutational analysis of the yeast RNA helicase Sub2p reveals conserved domains required for growth, mRNA export, and genomic stability. RNA.

[67] Begley TJ, Rosenbach AS, Ideker T, Samson LD (2002). Damage recovery pathways in Saccharomyces cerevisiae revealed by genomic phenotyping and interactome mapping. Mol Cancer Res.

[68] Begley TJ, Rosenbach AS, Ideker T, Samson LD (2004). Hot spots for modulating toxicity identified by genomic phenotyping and localization mapping. Mol Cell.

[69] Bennett CB, Lewis LK, Karthikeyan G, Lobachev KS, Jin YH, Sterling JF, Snipe JR, Resnick MA (2001). Genes required for ionizing radiation resistance in yeast. Nat Genet.

[70] Wilson DM, Deutsch WA, Kelley MR (1994). Drosophila ribosomal protein S3 contains an activity that cleaves DNA at apurinic/apyrimidinic sites. J Biol Chem.

[71] Yacoub A, Kelley MR, Deutsch WA (1996). Drosophila ribosomal protein PO contains apurinic/apyrimidinic endonuclease activity. Nucleic Acids Res.

[72] Chai Q, Zheng L, Zhou M, Turchi JJ, Shen B (2003). Interaction and stimulation of human FEN-1 nuclease activities by heterogeneous nuclear ribonucleoprotein A1 in alpha-segment processing during Okazaki fragment maturation. Biochemistry.

[73] Xiao R, Sun Y, Ding JH, Lin S, Rose DW, Rosenfeld MG, Fu XD, Li X (2007). Splicing regulator SC35 is essential for genomic stability and cell proliferation during mammalian organogenesis. Mol Cell Biol.

[74] Li X, Manley JL (2005). Inactivation of the SR protein splicing factor ASF/SF2 results in genomic instability. Cell.

[75] Kim YJ, Wilson DM (2012). Overview of base excision repair biochemistry. Curr Mol Pharmacol.

[76] Dalhus B, Laerdahl JK, Backe PH, Bjoras M (2009). DNA base repair—recognition and initiation of catalysis. FEMS Microbiol Rev.

[77] Luna L, Rolseth V, Hildrestrand GA, Otterlei M, Dantzer F, Bjoras M, Seeberg E (2005). Dynamic relocalization of hOGG1 during the cell cycle is disrupted in cells harbouring the hOGG1-Cys326 polymorphic variant. Nucleic Acids Res.

[78] Morland I, Rolseth V, Luna L, Rognes T, Bjoras M, Seeberg E (2002). Human DNA glycosylases of the bacterial Fpg/MutM superfamily: an alternative pathway for the repair of 8-oxoguanine and other oxidation products in DNA. Nucleic Acids Res.

[79] Bravard A, Campalans A, Vacher M, Gouget B, Levalois C, Chevillard S, Radicella JP (2010). Inactivation by oxidation and recruitment into stress granules of hOGG1 but not APE1 in human cells exposed to sub-lethal concentrations of cadmium. Mutat Res.

[80] Chattopadhyay R, Das S, Maiti AK, Boldogh I, Xie J, Hazra TK, Kohno K, Mitra S, Bhakat KK (2008). Regulatory role of human AP-endonuclease (APE1/Ref-1) in YB-1-mediated activation of the multidrug resistance gene MDR1. Mol Cell Biol.

[81] Marenstein DR, Ocampo MT, Chan MK, Altamirano A, Basu AK, Boorstein RJ, Cunningham RP, Teebor GW (2001). Stimulation of human endonuclease III by Y box-binding protein 1 (DNA-binding protein B). Interaction between a base excision repair enzyme and a transcription factor. J Biol Chem.

[82] Das S, Chattopadhyay R, Bhakat KK, Boldogh I, Kohno K, Prasad R, Wilson SH, Hazra TK (2007). Stimulation of NEIL2-mediated oxidized base excision repair via YB-1 interaction during oxidative stress. J Biol Chem.

[83] Abate C, Patel L, Rauscher FJ, Curran T (1990). Redox regulation of fos and jun DNA-binding activity in vitro. Science.

[84] Xanthoudakis S, Miao G, Wang F, Pan YC, Curran T (1992). Redox activation of Fos-Jun DNA binding activity is mediated by a DNA repair enzyme. EMBO J.

[85] Okazaki T, Chung U, Nishishita T, Ebisu S, Usuda S, Mishiro S, Xanthoudakis S, Igarashi T, Ogata E (1994). A redox factor protein, ref1, is involved in negative gene regulation by extracellular calcium. J Biol Chem.

[86] Robson CN, Milne AM, Pappin DJ, Hickson ID (1991). Isolation of cDNA clones encoding an enzyme from bovine cells that repairs oxidative DNA damage in vitro: homology with bacterial repair enzymes. Nucleic Acids Res.

[87] Wilson DM, Takeshita M, Grollman AP, Demple B (1995). Incision activity of human apurinic endonuclease (Ape) at abasic site analogs in DNA. J Biol Chem.

[88] Chen DS, Herman T, Demple B (1991). Two distinct human DNA diesterases that hydrolyze 3′-blocking deoxyribose fragments from oxidized DNA. Nucleic Acids Res.

[89] Johnson AW, Demple B (1988). Yeast DNA 3′-repair diesterase is the major cellular apurinic/apyrimidinic endonuclease: substrate specificity and kinetics. J Biol Chem.

[90] Xanthoudakis S, Smeyne RJ, Wallace JD, Curran T (1996). The redox/DNA repair protein, Ref-1, is essential for early embryonic development in mice. Proc Natl Acad Sci USA.

[91] Chattopadhyay R, Wiederhold L, Szczesny B, Boldogh I, Hazra TK, Izumi T, Mitra S (2006). Identification and characterization of mitochondrial abasic (AP)-endonuclease in mammalian cells. Nucleic Acids Res.

[92] Fan Z, Beresford PJ, Zhang D, Xu Z, Novina CD, Yoshida A, Pommier Y, Lieberman J (2003). Cleaving the oxidative repair protein Ape1 enhances cell death mediated by granzyme A. Nat Immunol.

[93] Berquist BR, McNeill DR, Wilson DM (2008). Characterization of abasic endonuclease activity of human Ape1 on alternative substrates, as well as effects of ATP and sequence context on AP site incision. J Mol Biol.

[94] Barnes T, Kim WC, Mantha AK, Kim SE, Izumi T, Mitra S, Lee CH (2009). Identification of Apurinic/apyrimidinic endonuclease 1 (APE1) as the endoribonuclease that cleaves c-myc mRNA. Nucleic Acids Res.

[95] Vascotto C, Fantini D, Romanello M, Cesaratto L, Deganuto M, Leonardi A, Radicella JP, Kelley MR, D’Ambrosio C, Scaloni A, Quadrifoglio F, Tell G (2009). APE1/Ref-1 interacts with NPM1 within nucleoli and plays a role in the rRNA quality control process. Mol Cell Biol.

[96] Tell G, Wilson DM, Lee CH (2010). Intrusion of a DNA repair protein in the RNome world: is this the beginning of a new era?. Mol Cell Biol.

[97] Antoniali G, Lirussi L, Poletto M, Tell G (2013). Emerging roles of the nucleolus in regulating the DNA damage response: the noncanonical DNA repair enzyme APE1/Ref-1 as a paradigmatical example. Antioxid Redox Signal.

[98] Haushalter KA, Stukenberg PT, Kirschner MW, Verdine GL (1999). Identification of a new uracil-DNA glycosylase family by expression cloning using synthetic inhibitors. Curr Biol.

[99] Kavli B, Sundheim O, Akbari M, Otterlei M, Nilsen H, Skorpen F, Aas PA, Hagen L, Krokan HE, Slupphaug G (2002). hUNG2 is the major repair enzyme for removal of uracil from U: a matches, U: G mismatches, and U in single-stranded DNA, with hSMUG1 as a broad specificity backup. J Biol Chem.

[100] Wibley JE, Waters TR, Haushalter K, Verdine GL, Pearl LH (2003). Structure and specificity of the vertebrate anti-mutator uracil-DNA glycosylase SMUG1. Mol Cell.

[101] Matsubara M, Tanaka T, Terato H, Ohmae E, Izumi S, Katayanagi K, Ide H (2004). Mutational analysis of the damage-recognition and catalytic mechanism of human SMUG1 DNA glycosylase. Nucleic Acids Res.

[102] Masaoka A, Matsubara M, Hasegawa R, Tanaka T, Kurisu S, Terato H, Ohyama Y, Karino N, Matsuda A, Ide H (2003). Mammalian 5-formyluracil-DNA glycosylase. 2. Role of SMUG1 uracil-DNA glycosylase in repair of 5-formyluracil and other oxidized and deaminated base lesions. Biochemistry.

[103] Mi R, Dong L, Kaulgud T, Hackett KW, Dominy BN, Cao W (2009). Insights from xanthine and uracil DNA glycosylase activities of bacterial and human SMUG1: switching SMUG1 to UDG. J Mol Biol.

[104] Kemmerich K, Dingler FA, Rada C, Neuberger MS (2012). Germline ablation of SMUG1 DNA glycosylase causes loss of 5-hydroxymethyluracil- and UNG-backup uracil-excision activities and increases cancer predisposition of Ung-/-Msh2-/- mice. Nucleic Acids Res.

[105] Nilsen H, Haushalter KA, Robins P, Barnes DE, Verdine GL, Lindahl T (2001). Excision of deaminated cytosine from the vertebrate genome: role of the SMUG1 uracil-DNA glycosylase. EMBO J.

[106] An Q, Robins P, Lindahl T, Barnes DE (2005). C – > T mutagenesis and gamma-radiation sensitivity due to deficiency in the Smug1 and Ung DNA glycosylases. EMBO J.

[107] Jobert L, Skjeldam HK, Dalhus B, Galashevskaya A, Vagbo CB, Bjoras M, Nilsen H (2013). The human base excision repair enzyme SMUG1 directly interacts with DKC1 and contributes to RNA quality control. Mol Cell.

[108] Hoskins J, Butler JS (2008). RNA-based 5-fluorouracil toxicity requires the pseudouridylation activity of Cbf5p. Genetics.

[109] Pettersen HS, Visnes T, Vagbo CB, Svaasand EK, Doseth B, Slupphaug G, Kavli B, Krokan HE (2011). UNG-initiated base excision repair is the major repair route for 5-fluorouracil in DNA, but 5-fluorouracil cytotoxicity depends mainly on RNA incorporation. Nucleic Acids Res.

[110] Rácz I. KIaLD (1978) Effect of light on the nucleotide composition of rRNA of wheat seedlings. Planta 142(3):263–267 10.1007/BF00385075 24408187

[111] Hoskins J, Scott Butler J (2007). Evidence for distinct DNA- and RNA-based mechanisms of 5-fluorouracil cytotoxicity in Saccharomyces cerevisiae. Yeast (Chichester, England).

[112] Sengupta T, Torgersen ML, Kassahun H, Vellai T, Simonsen A, Nilsen H (2013). Base excision repair AP endonucleases and mismatch repair act together to induce checkpoint-mediated autophagy. Nat Commun.

[113] Petersen-Mahrt SK, Neuberger MS (2003). In vitro deamination of cytosine to uracil in single-stranded DNA by apolipoprotein B editing complex catalytic subunit 1 (APOBEC1). J Biol Chem.

[114] Guo Z, Qian L, Liu R, Dai H, Zhou M, Zheng L, Shen B (2008). Nucleolar localization and dynamic roles of flap endonuclease 1 in ribosomal DNA replication and damage repair. Mol Cell Biol.

